# Mathematical modeling of evolution of cell networks in epithelial tissues

**DOI:** 10.1002/qub2.62

**Published:** 2024-07-07

**Authors:** Ivan Krasnyakov

**Affiliations:** ^1^ Department of Applied Physics Perm National Research Polytechnic University Perm Russia

**Keywords:** bubble‐like cells, growth epithelial tissue, mathematical model, tissue modeling, vertex model

## Abstract

Epithelial cell networks imply a packing geometry characterized by various cell shapes and distributions in terms of number of cell neighbors and areas. Despite such simple characteristics describing cell sheets, the formation of bubble‐like cells during the morphogenesis of epithelial tissues remains poorly understood. This study proposes a topological mathematical model of morphogenesis in a squamous epithelial. We introduce a new potential that takes into account not only the elasticity of cell perimeter and area but also the elasticity of their internal angles. Additionally, we incorporate an integral equation for chemical signaling, allowing us to consider chemo‐mechanical cell interactions. In addition to the listed factors, the model takes into account essential processes in real epithelial, such as cell proliferation and intercalation. The presented mathematical model has yielded novel insights into the packing of epithelial sheets. It has been found that there are two main states: one consists of cells of the same size, and the other consists of “bubble” cells. An example is provided of the possibility of accounting for chemo‐mechanical interactions in a multicellular environment. The introduction of a parameter determining the flexibility of cell shapes enables the modeling of more complex cell behaviors, such as considering change of cell phenotype. The developed mathematical model of morphogenesis of squamous epithelium allows progress in understanding the processes of formation of cell networks. The results obtained from mathematical modeling are of significant importance for understanding the mechanisms of morphogenesis and development of epithelial tissues. Additionally, the obtained results can be applied in developing methods to influence morphogenetic processes in medical applications.

## 1 INTRODUCTION

In the last decade, the number of studies dedicated to the development of mathematical models of behavior in living multicellular tissues has increased [1, 2, 3, 4, 5, 6]. This interest among scientists is associated with the rapid advancement of computer technologies, which enable in silico investigations without harm to real living organisms. Furthermore, mathematical models allow for tracking the evolutionary dynamics of a system, which is difficult to achieve using experimental research methods.

There are two approaches to developing complex multicellular system models—continuous and discrete. The most suitable approach for modeling the development of epithelial tissues is the discrete one, based on the vertex model. The vertex model is a valuable mathematical model for describing the dynamics of epithelial cell layers. This is supported by the popularity of using such models [1, 2, 5, 7, 8, 9, 10, 11, 12]. In these models, a cell is represented as a collection of vertices connected by edges or links. These models focus on simulating the internal structure of cells and their interactions with neighboring cells. Vertices can be mobile and deform under the influence of external forces or interactions with other cells. This type of model is widely used to study the mechanics of epithelial tissues, organ formation and development, embryogenesis, and other biological processes where the analysis and modeling of cell interactions are crucial. In the work [2], an overview of various vertex models for multicellular tissues is presented. One significant advantage of this type of model over others is that the interaction of elements in the entire system determines the potential. The terms that make up the potential can be easily interpreted from a physical point of view. An important feature of this approach is that it allows for the most realistic modeling of the cell, which is the elementary unit of living multicellular tissue. For example, in the work [1], the authors demonstrated that cell elongation induced by anisotropic stress affects cell proliferation rate, cell division orientation, and tension in cell junctions, all exerting different influences on the morphology of compartment boundaries during tissue growth. The authors found that global tissue anisotropy promotes the formation of boundaries in cell networks. In addition, in Ref. [13], a vertex model of morphogenesis was developed. It was used to compare with a natural experiment of epithelial cell network rearrangement in the wing of a *Drosophila*. In the work [12], the authors present a new formulation of such a model, which separately considers the force of contraction and the force of adhesion between cells. This work studied the stretching of a cell sheet by external forces, providing insights into cell remodeling due to stretching. In another study [14], the authors investigated the chemo‐mechanical interactions between cells. The modeling results showed that even a simple, elementary connection between chemical signal transmission and cell mechanics can generate a variety of patterns in epithelial tissues. Under certain parametric conditions, bimodal distributions of cell size and shape are obtained.

Epithelial cell networks exhibit a packing geometry characterized by various cell shapes, variations in the number of neighbors, and areas. The packing geometry is determined by developmental mechanisms that likely control the biophysical properties of cells and their interactions. Accounting for changes in the structural or material properties of cells and tissues, such as phase transitions, with microscopic interactions of their components, is a complex task at both experimental and theoretical levels. Furthermore, packing geometry is rigorously controlled during morphogenesis. For instance, in the wing epithelium of *Drosophila*, cells transform from irregular shapes to hexagonal shapes shortly before the formation of hair follicles [15].

In the literature dedicated to in vivo studies, a cellular network with “bubble” cells is often observed, yet authors leave this phenomenon unexplained. For example, in the study [16], the authors observed a cell network consisting of cells of different diameters in Hodgkin’s lymphoma. It is worth noting that cancerogenesis is a specific case of morphogenesis. In works [15, 17], which focused on studying the mechanisms of embryonic epithelial tissue in *Drosophila* during morphogenesis, a similar pattern of epithelial networks with bubble‐like cells was observed. The first study [15] investigated cell behavior that governs the morphogenesis of the follicular epithelium of *Drosophila* during early‐stage expansion and elongation of egg chambers. The second study [17] explored the mechanical aspects of the developing epithelial wing sheet of the *Drosophila*. A relatively similar pattern is also observed in the work [18], which mentions the self‐assembly of bubbles in the eye of the *Drosophila*. Additionally, in the study [19], which demonstrated the development of the zebrafish embryo’s eye, a cell network resembling bubble‐like cells is observed.

Despite the abundance and diversity of studies using vertex models, mention of cellular sheets with bubble‐like cells is rare in the literature. In one modeling study [14], bubble‐like cells were observed, yet their formation was noted during the remodeling process of mature epithelial tissues due to chemo‐mechanical interactions. In another investigation [20], bubble‐like cells were also observed, but in this study, cell division was organized in such a way that the plane of division was chosen randomly. A dividing cell in this manner may significantly influence the emergence of bubble‐like cells. In addition, the formation of bubble‐like cells is due to additional settings of the mathematical model.

The paper presents a chemo‐mechanical mathematical model of epithelial tissue morphogenesis. Special attention is paid to the structural unit of multicellular tissue, the cell, as an element of a large complex system during modeling. The cell division process in the model is implemented symmetrically, similar to what occurs in healthy cells. The model presented differs from those in the literature due to the introduced potential. The potential we introduced takes into account not only the elasticity of cell perimeter and area but also the elasticity of cell internal angles. The study includes an investigation of the morphological forms of cell networks that arise during the evolution of epithelial tissue. Various configurations of cell networks (cells of equal sizes, bubble‐like cells, and spindle‐like shape cells) during epithelial tissue morphogenesis were obtained and thoroughly analyzed.

## 2 RESULTS

Here we briefly present the main equation of the mathematical model. Cells in the model are represented as polygons. In solving our problem, the most suitable cell shape is a regular hexagon. To model the dynamics of the entire system, we define the equation for potential energy as follows:

(1) $$U\left(t\right)=\sum_{i=1}^{N_{c}}\left[\eta {\left(A_{i}-A_{0}\right)}^{2}+\mu P_{i}^{2}+\sigma \sum_{j=1}^{n_{0}}{\left(l_{ij}-l_{0}\right)}^{2}+\phi \sum_{j=1}^{n_{0}}{\left(\alpha_{ij}-\alpha_{0}\right)}^{2}\right].$$

Here, the first term describes the elasticity of the cell area with the elastic coefficient *η*. Where, *A*_*i*_ represents the current area of the cell, and *A*_0_ is the average value of the area for all cells, calculated using the formula:

(2) $$A_{0}=\frac{3\sqrt{3}}{2}a,$$

where *a* is the length of an edge of a regular hexagon. The value of the area in the model is calculated using the formula:

(3) $$A_{i}=\frac{1}{2}\left|\sum_{j=1}^{n_{0}}\left[\left(x_{j}+x_{j+1}\right)\cdot \left(y_{j}-y_{j+1}\right)\right]\right|,\text{where}\;\left(x_{n_{0}+1},y_{n_{0}+1}\right)=\left(x_{1},y_{1}\right).$$

The second term in Equation (1) describes the contractility of the cell perimeter with the coefficient *μ*, which can be interpreted as the contractility of the cell cytoskeleton. The third term represents the elasticity of the cell edges, where *σ* is the elasticity coefficient of the edge, *l*_*ij*_ is the length of the *j*th edge of the *i*th cell, and *l*_0_ is the average edge length, equal to the length of an edge of a regular hexagon (*l*_0_ = *a*, see Section 5.1. Mathematical Model). The fourth term in Equation (1) describes the elasticity of the cell’s rounded shape with the coefficient *ϕ*, where *α*_*ij*_ is the current value of the *j*th angle of the *i*th cell in radians, calculated using the formula:

(4) $$\alpha_{ij}=\arccos\left(\cos\left(\alpha_{ij}\right)\right),\;\cos\left(\alpha_{ij}\right)=\frac{\left(b,c\right)}{\left|b|\cdot |c\right|},$$

and *α*_0_ is the average angle value (*α*_0_ = π/3). In most cases, cells have a rounded shape, and only in certain states can they have negative angles [21]. Therefore, we assume that the coefficient *ϕ* = 1, which means that the deviations of the cell’s internal angle *α*_*ij*_ from the average value *α*_0_ are minimal. By doing so, we limit the elasticity of polygonal cells to prevent the occurrence of polygons with negative angles.

Summation in Equation (1) in the third and fourth terms is done over the number of sides of the polygonal cell, which is denoted as *n*_0_. The overall summation is performed over all cells in the system, denoted as *N*_*c*_. It is worth noting that the number of sides of the polygon is a dynamically changing quantity, *n*_0_(*t*), as is the total number of cells in the system, *N*_*c*_(*t*).

Before studying tissue morphology, let us first discuss some general properties of the model. An important feature is the primary states, or the most relaxed configurations of the cell network, which correspond to the minimum potential energy of the entire system. However, these states do not correspond to realistic tissue morphology, which will be discussed further below. Yet, the primary states serve as reference states when all cells are identical, that is, *A*_*i*_ = *A*_0_, *l*_*ij*_ = *l*_0_, *α*_*ij*_ = *α*_0_. Next, the results of a detailed investigation of Equation (1) will be presented, which describe the potential of the entire cellular tissue system in our mathematical model.

### 2.1 Case 1

**FIGURE 1:**
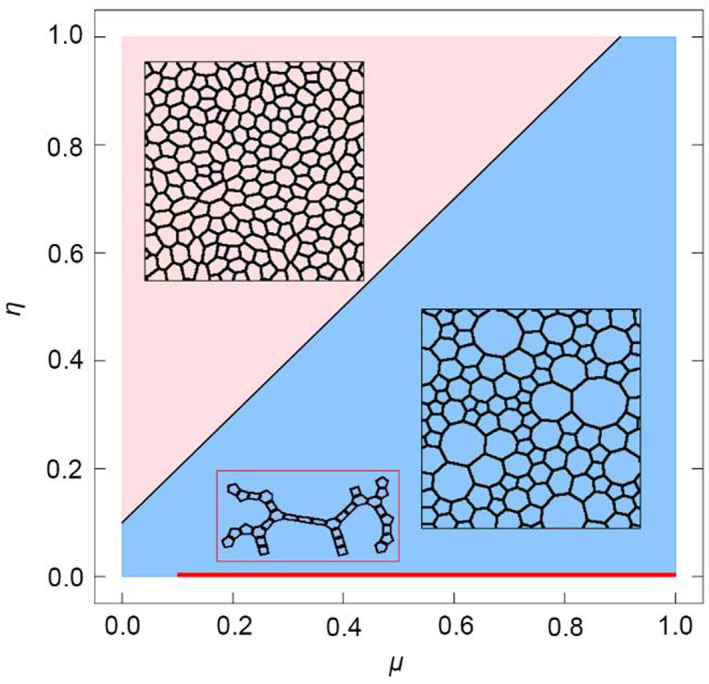
Diagram of the main states of the model. Fixed *σ* = 1, *ϕ* = 1, and varying parameters *μ* and *η*.

Figure 1 shows the primary configurations of the cell network, where the following regions can be distinguished: pink—cells of the same size; blue—presence of “bubble” cells; and red—thread‐like tissue shape. These geometries of the cell network are determined by two parameters of Equation (1): *μ*—contractility and *η*—elasticity. The parameter *ϕ = *1 means that deviations of the internal cell angle *α*_*ij*_ from the average value *α*_0_ are minimal, and the edge elasticity coefficient in this case takes the value *σ* = 1. This indicates that the cell edges are practically incompressible, and their length tends to the equilibrium value *l*_0_.

All calculations were performed with the same initial conditions—the tissue consists of 32 cells with free boundaries. The parameters were changed in increments Δ*η* and Δ*μ = *0.1. Let us examine the process of tissue development in different main states highlighted in the diagram (Figure 1) in more detail.

**FIGURE 2:**
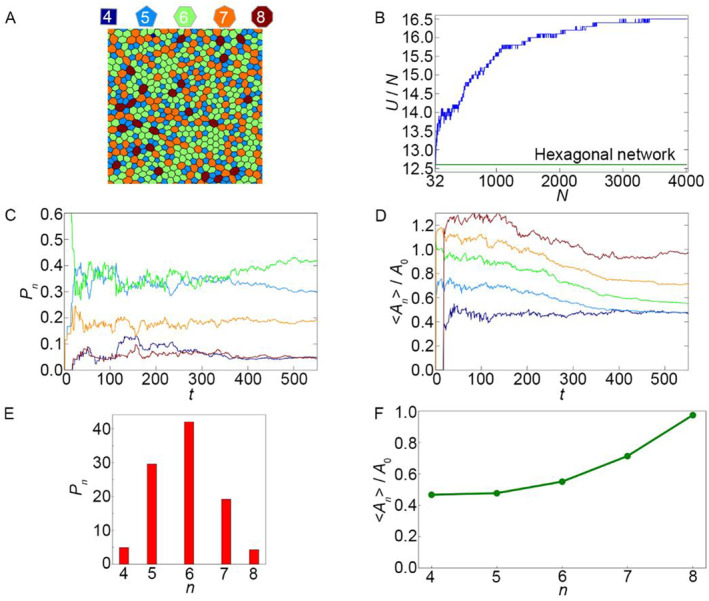
Case, corresponding to a point in the pink region of the state diagram (Figure 1), parameters *μ* = 0.1 and *η* = 0.9. (A) Image of the polygonal mesh of the cell tissue. (B) Energy per cell *U*/*N*. (C) Fraction of cells with different polygonal shapes in the tissue *P*_*n*_(*t*). (D) Normalized average area of cells with different numbers of vertices <*A*_*n*_>(*t*)/*A*_0_. (E) Steady‐state value of *P*_*n*_ for different polygonal classes. (F) Steady‐state value of <*A*_*n*_>/*A*_0_ for different polygonal classes.

The case where cells of approximately equal sizes are formed (Figure 1, pink region) occurs when the pair of *η*–*μ* values is located above the black line. Figure 2A shows the state of the cell network in this configuration. The simulation results demonstrate that as the number of cells increases, the energy per cell (*U*/*N*) (Figure 2B) also increases. The initial stage of tissue growth is accompanied by a sharp increase in energy, and with further increases in the number of cells, the energy growth slows down, tending toward a certain constant value that exceeds the value of the main state (green line in Figure 2B) of the undeformed hexagonal lattice of cells.

During this process, a pattern in the form of a polygon lattice (Figure 2A) emerges, which can be characterized by the fraction of cells with different polygon shapes (i.e., cells with different numbers of vertices *n*), denoted as *P*_*n*_(*t*), and the normalized values of the average area of cells with *n* vertices <*A*_*n*_>(*t*) to their average area value *A*_0_. At the initial time point, the fraction of *n*‐sided cells *P*_*n*_(*t*) (Figure 2C) and their normalized average area <*A*_*n*_>(*t*)/*A*_0_ (Figure 2D) are close to unity. This can be attributed to the initial configuration consisting of 32 undeformed hexagonal cells. However, as the system evolves, these quantities start to fluctuate and eventually converge to stationary values. When we refer to octagonal cells, it also includes cells‐polygons with a number of sides *n*_0_ > 8.

From the plots in Figure 2C,D, it can be inferred that as the number of cells increases, the tissue network pattern becomes stationary. The steady‐state values of *P*_*n*_ and <*A*_*n*_>/*A*_0_ for different polygon classes are shown in Figure 2E,F, respectively. It can be observed that for the network at the final time point, hexagons are the most common polygon class, and the average number of sides of neighboring polygonal cells is <*n*> = 6.38. The normalized average area <*A*_*n*_>(*t*)/*A*_0_ for quadrilateral cells fluctuates around a constant value (Figure 2D), while for pentagonal, hexagonal, heptagonal, and octagonal cells, it decreases over time and reaches a steady‐state value. This observed effect is related to the fact that quadrilateral cells appear as a result of division of pentagonal cells or intercalation. The lower the number of sides a cell has, the lower the probability of its division, which is determined by the distribution (7). Regarding hexagonal cells, it can be seen that their normalized area deviates from the average value *A*_0_ (Figure 2F) by approximately 40%. This is consistent with experimental observations of epithelial cell division in zebrafish embryo [22]. A similar trend is observed for pentagonal, heptagonal, and octagonal cells.

In another case (Figure 1, blue region), the formation of “bubble” cells occurs. Such a pattern of the cell network is formed when the pair of values *η* – *μ* is below the black line. Figure S1A shows the state of the cell network. The obtained cell network is observed in real experiments with epithelial tissues [17, 19]. It is worth noting that similar cell networks are observed in developing tissues. In developed tissues, a cell network is observed, as shown in Figure 1 (pink region). As the number of cells increases, the energy per cell initially decreases sharply and then begins to increase (Figure S1B). With further increases in the number of cells, the growth of system energy slows down and reaches a steady state, which does not exceed the value of the hexagonal lattice of undeformed cells (green line, Figure S1B).

The “bubble” cells that form during tissue evolution (Figure S1A) are cells with a number of edges *n*_0_ ≥ 7. This type of network exhibits similarities to case 1 in the distribution of cells with different numbers of sides (see Figures 2B and S1B, Figures 2D and S1D). It can be observed that in this case, the number of quadrilateral cells sharply increases initially and then decreases. From the graph of normalized average area of cells <*A*_*n*_>(*t*)/*A*_0_ (Figure S1D), a qualitative similarity to case 1 (Figure 2D) is evident, but the quantitative data differ significantly. In this case the average area of quadrilateral cells is approximately <*A* ^4^> ≈ 0.25*A*_0_ (Figure S1F), while for hexagonal cells, which are considered as standard within the model, it is <*A* ^6^> ≈ 0.5 *A*_0_. This behavior of the system is due to the dominance of the second term over the first term in Equation (1). Thus, the perimeter of cells is reduced, leading to a decrease in their area. The reduction in perimeter is more significant for cells with fewer sides. Octagonal and higher‐sided cells do not deviate from the average value *A*_0_, while other polygonal cells contract. Similarly to case 1, in the final moment in time, hexagonal cells are the most common polygonal class, and the average number of sides for neighboring cells is <*n*> = 6.28 (Figure S1E).

**FIGURE 3:**
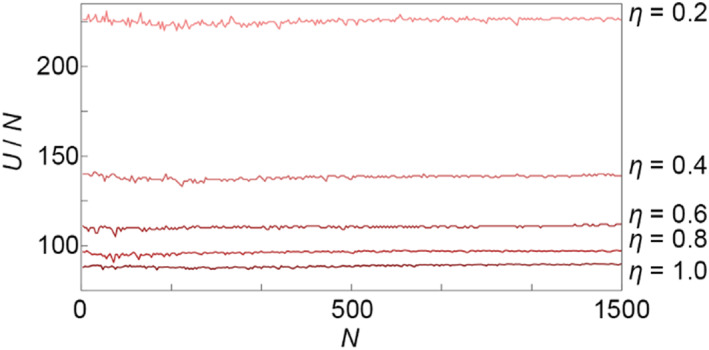
Energy per cell. Vertical slice of the diagram of the main states (Figure 1) at a fixed parameter *μ* = 0.5.

It is worth noting that the closer the pair of values *η*–*μ* is to the black boundary (Figure 1), the smaller the sizes of the “bubble” cells become. From this, we can conclude that increasing the coefficient *η* compensates for the deviation from the minimum potential energy of the system (Figure 3). The state of the system at *η = *1 corresponds to the minimum potential energy, which means it is more energetically favorable.

**FIGURE 4:**
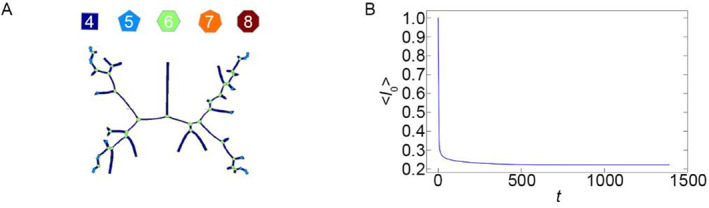
Case, corresponding to a point in the red region of the state diagram (Figure 1), parameters *μ* = 0.4 and *η* = 0.0. (A) Image of the polygonal mesh of the cell tissue. (B) The average edge length of a cell <*l*_0_>(*t*).

In the third case (Figure 1, red region), the formation of filamentous cell structures (Figure 4A) is observed, where cells with four sides are present. This effect is associated with the absence of the elasticity coefficient *η* = 0 in this case, meaning there is no term that influences the preservation of cell area, while the term with the coefficient *μ* fully affects the cell shape. If *μ* > 0, the cells tend to contract, and if *μ < *0, the cell boundaries expand. As can be seen from the graph of the average edge length of cells <*l*_0_>(*t*) (Figure 4B), there is a sharp decrease in this value followed by reaching a stationary state. From this, it can be concluded that the second term in Equation (1) dominates over the third term, leading to this effect. All of this indicates a sharp decrease in the energy of the system followed by reaching a constant value.

### 2.2 Case 2

**FIGURE 5:**
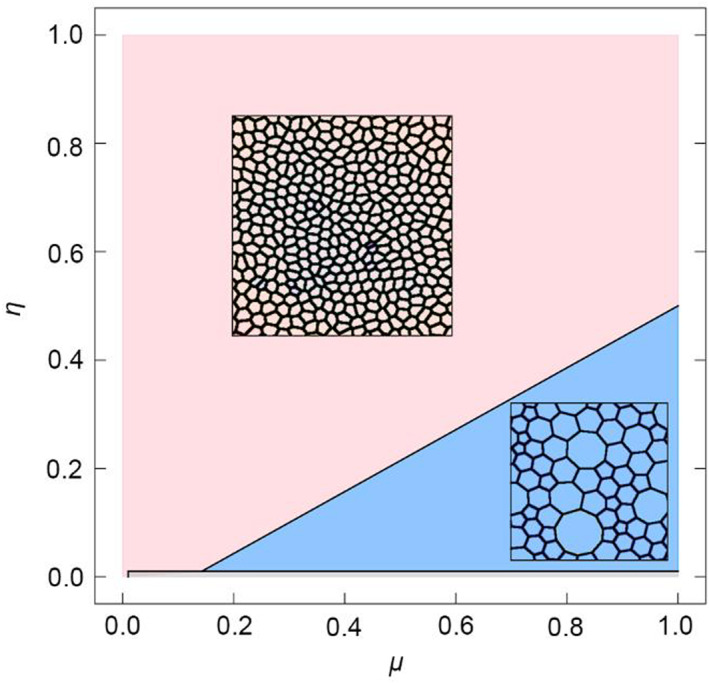
Diagram of the main states of the model. Fixed *σ* = 0, *ϕ* = 1 and varying parameters *μ* and *η*.

Let us consider the influence of the edge elasticity coefficient *σ* on the pattern of the cellular grid. When *σ* = 0, a phase diagram of the cellular grid’s main states was obtained (Figure 5), where the similar configurations as in the previous case 1 (Figure 1) are highlighted: pink—cells of equal sizes and blue—the presence of “bubble” cells. Instead of the red region (see Figure 1), a gray region appeared, which is structurally unstable. This means that for this pair of parameters *μ *–* η*, the model ceases to function during numerical calculations due to the emergence of topological defects. The edge elasticity coefficient *σ = *0 implies that the cell edges are fully compressible, and their length can freely deviate from the equilibrium value *l*_0_. It is noticeable how, with this parameter set, the blue region significantly decreased in the phase diagram. Let us discuss the main states in more detail.

**FIGURE 6:**
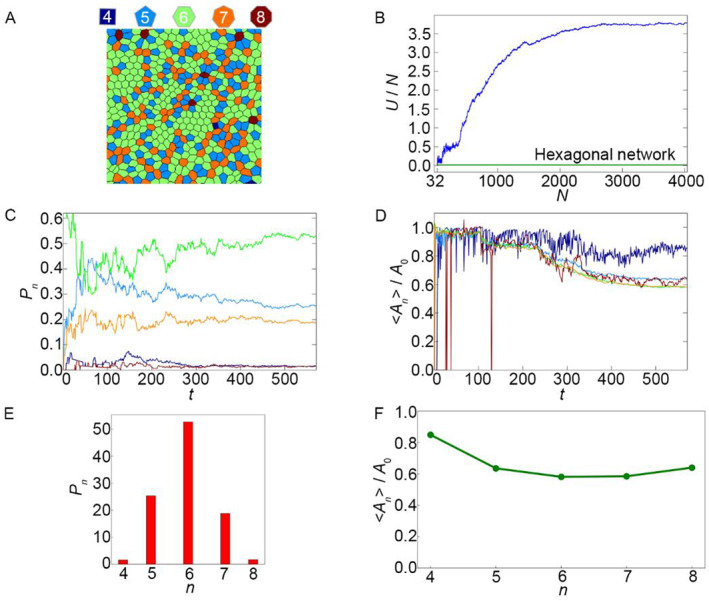
Case, corresponding to a point in the pink region of the state diagram (Figure 5), parameters *μ* = 0.0 and *η* = 1.0. (A) Image of the polygonal mesh of the cell tissue. (B) Energy per cell *U*/*N*. (C) Fraction of cells with different polygonal shapes in the tissue *P*_*n*_(*t*). (D) Normalized average area of cells with different numbers of vertices <*A*_*n*_>(*t*)/*A*_0_. (E) Steady‐state value of *P*_*n*_ for different polygonal classes. (F) Steady‐state value of <*A*_*n*_>/*A*_0_ for different polygonal classes.

In the pink region (Figure 5), we obtain the following pattern of the cellular grid (Figure 6A), characterized by the prevalence of hexagonal cells over other cell shapes (Figure 6C,D). At the beginning of the evolution of the cellular system, there is a sharp increase in the potential energy per cell (Figure 6B). When the system develops to *N* ≈ 2000 cells, the energy per cell (*U*/*N*) reaches a stationary value (Figure 6B). It should be noted that this value is much higher than the equilibrium state of undeformed hexagonal cells (green line in Figure 6B). From the graph of the normalized average cell area <*A*_*n*_>(*t*)/*A*_0_ (Figure 6D), it can be observed that throughout the evolution of the system, the sizes of cells with different numbers of vertices deviate from the average value *A*_0_, but not as significantly as in the previous case (Figures 2D and S1D). Polygonal cells with five or more sides deviate from *A*_0_ by approximately 40% (<*A* ^5‐8^> ≈ 0.6 *A*_0_), while quadrilateral cells deviate by 20% (<*A* ^4^> ≈ 0.8*A*_0_) (Figure 6F). The average number of sides of neighboring cells in the system is <*n*> = 6.42.

Let us compare this example with the example from the previous diagram of the main states (pink region, Figure 1). With *σ = *1, there is a prolonged competition between pentagonal and hexagonal cells (Figure 2C), which eventually results in the prevalence of hexagonal cells. In the system with *σ = *0 (Figure 6С), hexagonal cells become the dominant cell type almost from the beginning of the evolution. From the graph of the normalized average cell area <*A*_*n*_>(*t*)/*A*_0_ (Figure 2D), it can be observed how this change depends on the number of cell sides. At the established area values, it is evident that cells with a larger number of sides deviate from the average area *A*_0_ to a lesser extent compared to cells with smaller number of sides (Figure 2E). In this case with *σ = *0, it is noticeable how all cells deviate from the average value *A*_0_ by nearly the same magnitude (Figure 6D), except for quadrilateral cells, which have a smaller deviation. From the established tissue development regime, it can be seen that the distribution of cells by the number of sides also differs between *σ = *1 and *σ* = 0 (Figures 2E and 6E). The distribution in Figure 6E, with *σ = *0, shows that the number of hexagonal cells comprises slightly more than half of the total number of cells in the system, while in case 1 of *σ = *1, the quantity of hexagonal cells accounts for 40% of their total count (Figure 2E). This indicates that the simulation results, with the given pair of parameters *μ *–* η* and *σ* = 0, qualitatively and quantitatively resemble the behavior of real epithelial sheets [22, 23].

**FIGURE 7:**
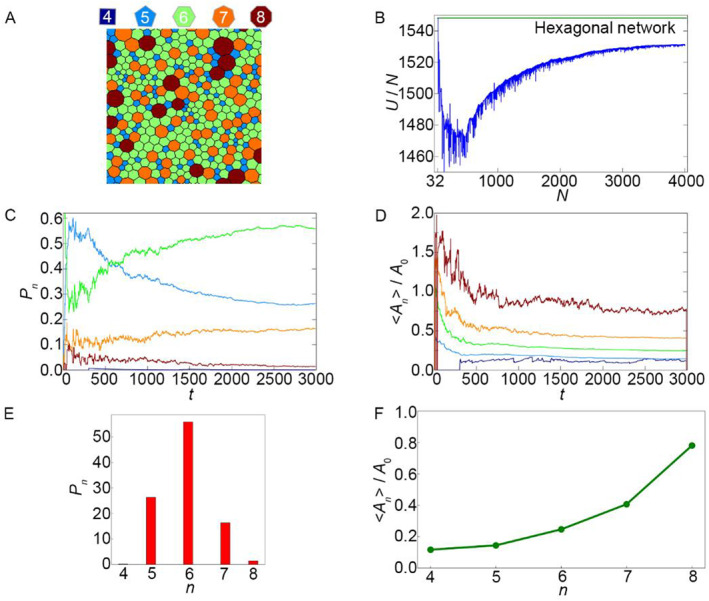
Case, corresponding to a point in the blue region of the state diagram (Figure 5), parameters *μ* = 1.0 and *η* = 0.1. (A) Image of the polygonal mesh of the cell tissue. (B) Energy per cell *U*/*N*. (C) Fraction of cells with different polygonal shapes in the tissue *P*_*n*_(*t*). (D) Normalized average area of cells with different numbers of vertices <*A*_*n*_>(*t*)/*A*_0_. (E) Steady‐state value of *P*_*n*_ for different polygonal classes. (F) Steady‐state value of <*A*_*n*_>/*A*_0_ for different polygonal classes.

Another main state of the cellular network (blue region in Figure 5) consists of cells of different sizes and shapes (Figure 7A). From the graph of energy per cell (Figure 7B), it can be observed that at the initial moment, the energy sharply decreases and reaches a minimum. Strong fluctuations are observed, followed by a monotonic increase and a gradual convergence to a constant value, which is lower than the energy of undeformed hexagonal cells (green line in Figure 7B). The graph (Figure 7C) is similar to the graph (Figure S1C) depicting the change in the proportion of cells of different shapes. This indicates that in both cases, with *σ = *1 and 0, tissue evolution in the blue region occurs in an identical manner. A similar pattern is observed when comparing the graphs of the normalized average area <*A*_*n*_>(*t*)/*A*_0_ (Figures 7D,F, S1D and S1F). This once again confirms the previous assumption about the similarity in tissue evolution in the blue region under different *σ* parameters. However, at longer development times when the value of *P*_*n*_ has already stabilized for each type of polygonal cell (Figure 7E), there is a clear predominance of hexagonal cells over cells of other shapes and the average number of sides per cell is <*n*> = 6.43.

It can be concluded that the parameter set *μ* – *η* from the pink region (Figure 5) is the most energetically favorable set compared to the identical parameter set from the pink region (Figure 1) and the blue regions (Figures 1 and 5). Analysis of the data shows that the *μ *–* η* parameter set in the pink region exhibits the most advantageous energy configuration compared to the other regions. This means that when using these parameters, the evolution of the cellular system leads to a more stable and energetically favorable structure. This conclusion is supported by the analysis of energy per cell, where the energy value for the pink region (Figure 5) is lower than that of the other regions, as well as by the analysis of cell shapes and sizes. The normalized average cell area and the distribution of the number of cell sides also indicate the advantages of this parameter set. Thus, selecting the *μ* – *η* parameters from the pink region (Figure 5) can be recommended for achieving a more stable and energetically favorable structure in epithelial sheets or similar systems.

### 2.3 Case 3

Figure S2 shows the result of the calculation with the variation of parameter *ϕ*, which affects the current value of the angle *α*_*ij*_ in the cells. In this case, *ϕ* was set to *ϕ = *0, which means that the angles in the cells can deviate freely from the mean value *α*_0_. This effect is well demonstrated in the cellular grid diagram (Figure S2A), where we can observe different types of internal cell angles: acute, obtuse, and negative angles. In this case, the energy per cell increases as follows: initially, there is a sharp increase, followed by a slight decrease, then the energy increases again, but less intensely, and there is also a decrease, followed by a monotonic growth and another small dissipation, until it reaches a constant value (Figure S2B). The values of the fraction of cells with different numbers of sides *P*_*n*_ (Figure S2C) in this system oscillate around their established values from the beginning of the evolution. The normalized cell area <*A*_*n*_>(*t*)/*A*_0_ (Figure S2D) has a negative trendline for each type of polygon, as in previous cases, and converges to a steady value closer to the end of the evolution. From the established fraction values of cells with different numbers of polygons (Figure S2E), it can be seen that in such a system, cells with five and six sides prevail, constituting approximately 70% of the total number of cells. Throughout the evolution of the system, there is competition among them (Figure S2C). It can be observed that the average area of quadrilateral cells is <*A* ^4^> ≈ 0.6*A*_0_ (Figure S2F); for hexagonal cells, which are the reference in the model, it is <*A* ^6^> ≈ 0.8*A*_0_, and for octagonal and higher‐sided cells, it is <*A* ^8^> ≈ 0.83*A*_0_.

Such a diagram of epithelial tissue cellular grid is not observed in natural biological systems because cells in the epithelial phenotype are not prone to such pronounced bending, forming negative internal angles. This shape is characteristic of cells that have undergone an epithelial–mesenchymal transition (EMT) and are in the mesenchymal phenotype [21]. Cells acquiring this phenotype begin to move within the tissue, exhibiting a high degree of flexibility between cells.

Currently, it can be said that the potential introduced by Equation (5), although simple, allows for modeling complex cell behavior. It is evident that introducing a chemo‐mechanical coupling for potential parameters is necessary when the task at hand is modeling the behavior of different cell groups in the epithelial tissue. It becomes clear that for modeling the behavior of cells in the epithelial phenotype, the parameter *ϕ* should take the value *ϕ* = 1, while for cells in the mesenchymal phenotype, this parameter should take values from the interval *ϕ* ϵ [0, 1).

### 2.4 Case 4

This section presents the results of modeling the chemo‐mechanical interaction of cells. Figure S3 illustrates the influence of a chemical signal on tissue mechanics, specifically the impact of the flow of actomyosin molecules on cell shape. The chemo‐mechanical interaction in the system was defined as follows:

(5) $$\phi =-k_{1}\left|C_{i}\right|+b_{1}.$$

**FIGURE 8:**
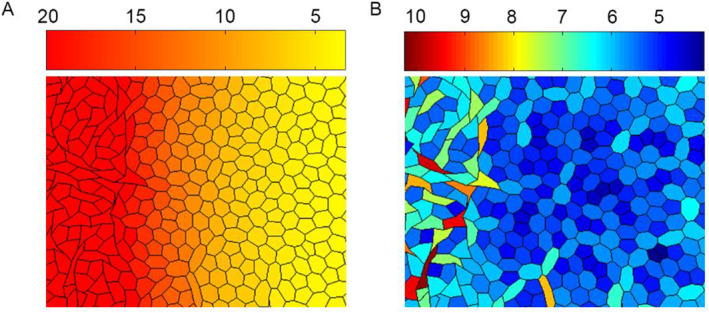
The fields of cellular tissue. (A) Field of chemical signal concentration in tissue. (B) Cells perimeter field.

Thus, the elastic coefficient *ϕ*, which regulates cell shape, is defined as a linear function dependent on the concentration of the chemical signal. The coefficients *k*_1_ and *b*_1_ are normalization parameters. A constant chemical signal source was applied at the left boundary of the computational domain. The field of the chemical signal concentration is depicted in Figure 8A. From the perimeter field (Figure 8B), it can be observed how cells located in the region of increased chemical signal concentration have altered their shape. This phenomenon can be qualitatively compared to EMT [21].

Furthermore, it is evident that in this system, the proportion of pentagonal and octagonal cells has significantly increased (Figure S3C,E) compared to Case 2 (Figure 6C,E), and the average number of sides per cell is <*n*> = 5.85.

Similar cellular networks have also been observed in experiments related to epithelial healing [24]. The authors of this study identified a key determinant of leader cell migration in wound healing. As noted by the authors, leading cells behave like “mechanical losers” [24]. They acquire a spindle‐like shape and undergo extrusion when compressed by the cells following them. From an energetic perspective, this means that leading cells have less energy than the cells behind them. So the system finds it more energetic and advantageous to extrude the leading cells.

**FIGURE 9:**
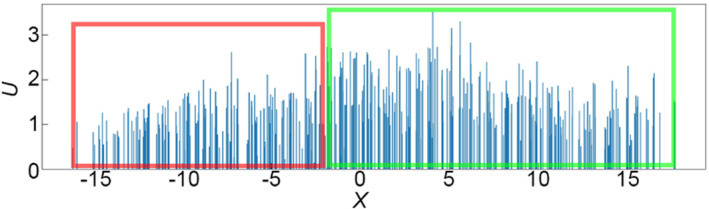
Distribution of potential energy in the tissue. Cross‐section along the computational domain along the *X*‐axis.

The simulation results reveal a similar pattern. In Figure 9, which depicts the distribution of potential energy in the epithelial tissue along the *X*‐axis cross‐section, for the case depicted in Figure 8. It is evident that cells located in the region of elevated chemical signal concentration have lower potential energy (red frame) compared to the cells behind them (green frame). Thus, the potential energy of hexagonal cells that are located inside transitions into kinetic energy of cell movement at the free boundary. It can be hypothesized that this effect contributes to the extrusion of leading cells, as observed in ref. [24].

## 3 DISCUSSION

This study is focused on exploring potential morphological forms of cell networks that arise during the collective evolution of epithelial tissues through mathematical modeling methods. To achieve this, a chemo‐mechanical mathematical model of the epithelial sheet was developed. Despite the fact that similar models have been described in detail in previous works [1, 2, 4, 7, 10, 11, 13, 14], we introduced a new potential that influences the morphology of cell networks during tissue development. Additionally, the model includes an integral equation of chemical signaling, allowing for the consideration of chemo‐mechanical interactions between cells.

We conducted a study on the evolution of a cellular system, representing epithelial tissue, using mathematical modeling methods. During the modeling of epithelial tissue morphogenesis, three different states of cell networks were identified.

The first state corresponds to a depicted cell network in which the cells are of equal size. This is the most common pattern of cell networks obtained both through mathematical modeling [1, 4, 13] and when studying tissues in vivo [13, 15]. This type of network has low potential energy. The distribution of cells in terms of shapes conforms to the normal distribution, with the most probable values for hexagonal cells [23, 25]. Additionally, the distribution of cell areas is in good agreement with experimental data [22] (see Figures 2 and 6).

The second state corresponds to a cell network with “bubble” cells. This morphology of cell networks is regularly encountered in experimental studies [16, 18, 19], but mentions of such cell networks in mathematical modeling studies are rare [14]. Cell networks with “bubble” cells (see Figures S1 and 7) are typically observed in tissues at their early developmental stages. When “bubble” cells appear in the tissue, its energetic state changes significantly. From an energetic perspective, the system becomes unstable, indicating the initiation of pathological processes in the tissue. Therefore, such systems require further investigation. The obtained results help establish a connection between cell size pleomorphic and epithelial cell packing control. For example, this may be relevant for understanding the spread of tumor cells in humans [14, 16]. Understanding the normal development of epithelial tissues helps in research and identifies anomalies and pathological conditions associated with these tissues, which is crucial for the diagnosis and treatment of various diseases. It is worth noting that this pattern is observed only in developing epithelial tissues. However, when this tissue reaches full development, bubble‐like cells are absent, and the cellular network pattern resembles what is shown in Figures 1 and 6 in the pink region (cells have the same size), for example, see Ref. [26]. Thus, the regions presented in the phase diagrams can be classified as developing and developed tissues.

In the third case, we obtain a cell network pattern in which the cells have a spindle‐like shape. Such cells are characterized by negative internal angles. These networks correspond to cells of a mesenchymal phenotype, that is, when they have undergone EMT [21]. Therefore, by introducing a new term into the potential Equation (1), we have been able to create a new mathematical model that allows us to simulate the development of epithelial sheets not only at different stages of their development but also to consider the transition of cell phenotypes.

In addition, the second state of the cellular network (blue area in Figures 1 and 5) can be compared with the molecular structure of the porous nanomaterial zeolite ZSM‐5. Comparing cellular systems to materials such as zeolites or other porous materials highlights the similarities in their structural characteristics. This analysis can aid in the development and creation of new nanomaterials with desired properties. Understanding the energetic configurations and stability of cellular systems can serve as an inspiration for creating materials with optimized porosity, surface area, and structural stability.

A region on the map of elastic parameters *μ *–* η* was identified where the evolving cellular system adopts the most favorable energetic and stable configuration. The findings from the study of cellular systems can also be applied to understanding the behavior and properties of epithelial tissues in organisms. Epithelial tissues play a crucial role in organ development, wound healing, and barrier functions [3]. Analyzing parameter sets and cellular structures provides insight into tissue morphology formation, cell shape, and overall stability. This knowledge can have practical applications in the fields of regenerative medicine [27], tissue engineering [28, 29], and understanding diseases related to epithelial tissues [10, 11, 30].

Cellular systems often exhibit self‐organizing properties, where complex structures and patterns emerge from simple interactions between individual cells. Studying energetic configurations and cell shape distributions allows for the exploration of self‐organization principles in biological systems. This knowledge can be applied in various fields, such as developmental biology, collective cell migration, and pattern formation in tissues and organs.

## 4 CONCLUSION

The developed mathematical model allows for modeling various types of cellular networks in squamous epithelial tissues (epithelial tissues of *Drosophila* wing [13], epithelial tissues of zebrafish embryo [22], monolayer epithelial tissues [31], malignant tissue formations [10, 32], phenotype change, cell migration [30, 33], etc.) by adjusting the elastic coefficients *μ*, *η*, *σ*, and *ϕ* in Equation (1).

Introducing the parameter *ϕ*, which determines the flexibility of cells and their freedom to deviate from the mean angle *α*_0_, allows for modeling more complex cell behaviors. This reflects the transition of cells into a mesenchymal phenotype, where cells become more mobile and capable of moving within the tissue between cells [21]. Although the developed mathematical model is simple, further investigation considering chemo‐mechanical couplings is necessary.

Overall, the analysis of cellular systems and their energetic configurations finds applications in various fields, including biomineralization, nanomaterials, tissue engineering, self‐organization in biology, and even materials science. By relating cellular systems to real‐world applications, the acquired knowledge can be applied to advance scientific understanding and the development of innovative technologies.

## 5 MATERIALS AND METHODS

### 5.1 Mathematical model

Before modeling the morphogenesis of the epithelial tissue, it is important to discuss its structure. The epithelial tissue is a collection of polarized, differentiated cells closely packed together in the form of a layer lying on the basal membrane. The epithelium forms the boundary between the external or internal environment of the organism and makes up a significant portion of glands. For example, the epithelium lines the skin’s surface, cornea of the eye, serous membranes, and inner surfaces of hollow organs in the digestive, respiratory, and urogenital systems. The epithelial tissue lacks intercellular substances, and the epithelial cells form a continuous layer located on the basal membrane through which they receive nourishment. Cells in tissues are connected to their neighbors by adhesion molecules along their shared surfaces and can interact with each other and the surrounding environment. These complex interactions can lead to significant morphogenetic deformations in developing tissues, such as folding, stretching, or narrowing, which play a crucial role in shaping the final form of multicellular organisms. Understanding how cells collectively solve this task is an important question at the intersection of physics, chemistry, developmental biology, and computer technology. It is worth noting that cells in the epithelium cannot move freely, which defines their phenotype as epithelial. This means that they are entirely governed by tissue‐level commands. In contrast, the mesenchymal phenotype of cells implies their ability to move freely within the epithelium. Such behavior is typical, for instance, in cancer cells that do not adhere to tissue‐level commands. Healthy cells can also transition to a mesenchymal phenotype, for example, during wound healing. Some cancer cells exhibit such an active behavior within tissues that is directly associated with microorganism behavior. This type of cell behavior is called ameboid. Such movement within the epithelial layer is characteristic of individual cancer cells, which can lead to tumor metastasis.

Internal cellular tensions in the epithelium result in effective forces that deform cell shapes. The cytoskeleton plays a major role in generating such internal tensions [34]. The actin cortex, in particular, generates surface tension and linear tension along cell membranes, which are mediated and balanced by adhesion between cells and the extracellular matrix [35, 36]. Throughout the development and adult stages, tissues are additionally subjected to mechanical influences from their surrounding environment. External structures, such as the basal membrane [37] or apical extracellular membrane [38], can mechanically constrain tissues.

An important process in the tissue is morphogenesis, which occurs when the cell phenotype changes (altering their mechanical properties), when cells divide or undergo apoptosis, or when the physical constraints imposed by the environment on the epithelium change. Understanding the physics of morphogenesis requires accounting for the laws of mechanics, whereby the forces acting in the tissue must be balanced. It is essential to comprehend that internal and external forces deforming the tissue are balanced by friction or viscous forces. Quantitative physical descriptions of tissues allow us to describe how the generation and balance of mechanical forces govern morphogenesis [39, 40].

Each cell in the system is represented as a polygon. As already known, there are three types of tessellations of the plane with regular polygons, namely triangles, squares, and hexagons [41]. In solving our problem, the most suitable cell shape is a regular hexagon. This polygon shape is energetically favorable as it approximates a circular shape. There is also a deeper reason rooted in topology. If each of the *N* cells in an infinite tiling has *Q* edges, then the total number of vertices is given as *V* = 1/3 *Q N*, and the total number of edges is *E* = 1/2 *Q N*. These numbers are related to the classical Euler’s theorem: *N* – *V *+ *E* = 1. Neglecting unity in the infinite tiling, we obtain *Q* = 6, which corresponds to a hexagonal lattice.

**FIGURE 10:**
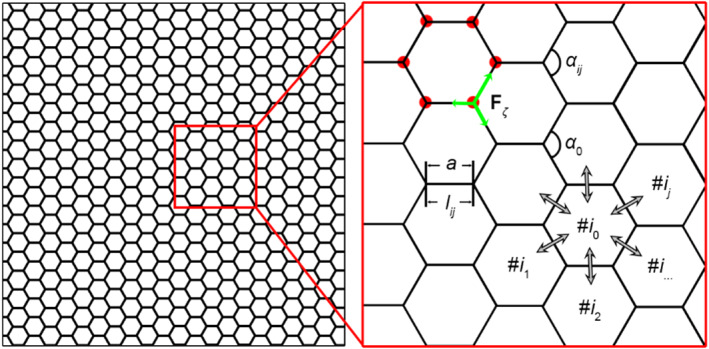
Schematic representation of the model cells. Forces (green arrows) act on the cell vertices marked with red dots.

The tissue evolves through the movement of the vertices (red dots in Figure 10) of polygonal cells. The mechanical force vector acting on a cell vertex (green arrows in Figure 10) is computed using the standard approach of potential force mechanics as the negative gradient of the potential energy with respect to the position vector of the *ζ*‐node.

(6) $$F_{\zeta }=-\frac{\partial U}{\partial R_{\zeta }}.$$

The cell’s movement speed is determined by the arithmetic mean of the velocities of its vertices. We formulate the vertex displacement equation within the framework of Aristotelian mechanics, where forces determine velocity rather than acceleration.

(7) $$V_{\zeta }=kF_{\zeta }H\left(\left|F_{\zeta }\right|-F_{0}\right).$$

Such an approach was chosen because the cellular tissue is a highly dissipative medium. This means that any movement of cells occurs without the inertia effect. For example, in the study by ref. [19], the authors demonstrated how an immune cell moves within epithelial tissue. They note that during cell movement, the speed often becomes zero or close to zero, with peak speeds reaching 10 μm/s. Additionally, it should be noted that epithelial cells are connected to each other by desmosomes and tightly adhere to one another. The movement of mesenchymal cells within the epithelium occurs with significant resistance due to the low permeability of the cellular environment, that is, without the inertia effect. A direct analogy can be drawn to the problem of fluid flow in a porous medium, where its infiltration happens with strong internal dissipation. In this case, fluid movement is described by Darcy’s equation, where there is no inertial term and the velocity of movement is directly determined by forces. This is the reason why the equation of motion was formulated within the framework of Aristotelian mechanics. Here, *k* is the mobility coefficient of the cell with appropriate dimensions, H is the Heaviside function, and *F*_0_ defines the critical force below which the node remains stationary.

An important mechanism of any living matter is its ability to self‐reproduce through cell division. In our model, we introduce a cell division mechanism that occurs with the following probability distribution.

(8) $$p_{\mathrm{div}}=p_{0}q^{n_{0}-6}.$$

**FIGURE 11:**
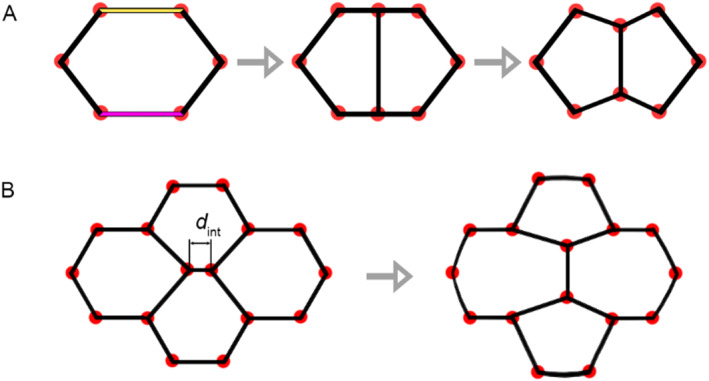
Schematic representation of cell division processes (A) and cell intercalation (B).

Here, *p*_0_ and *q* are parameters that determine the frequency of cell division, and *n*_0_ is the number of vertices in the cell. From Equation (8), it can be observed that when *q* ≥ 1, division is most likely to occur for cells with a number of vertices *n*_0_ ≥ 6. The cell division mechanism in the model is implemented as follows: (1) The longest edge of the cell is identified (marked in pink in the Figure 11A); (2) Its opposite edge is determined (marked in yellow in the Figure 11A) if the cell has an even number of edges. On the other hand, the larger of the two opposites is determined if the cell has an odd number of edges; (3) New nodes are placed at the centers of these two edges; (4) These nodes are connected by a new edge. The daughter cell is added to the end of the list and starts its evolution with instantaneous values of the physical and mechanical parameters of the mother cell. This algorithm allows for the simulation of mitotic cell division (Figure 11A).

Another equally important mechanism in living tissue is its cellular rearrangement in response to local excess stress. This mechanism is known as intercalation. It occurs whenever the length of the edge *l*_*ij*_ between neighboring cells becomes smaller than a critical value, *d*_int_ (Figure 11B). In simple terms, the bridge between the cells rotates by 90°.

In addition to the mechanical interactions between cells in the system, we introduce the exchange of chemical signals. The role of a chemical signal can be played by the concentration of oxygen, which is necessary for cells to maintain their viability. Additionally, there are processes of gene regulation that originate in the cell nucleus, for example, the synthesis of proteins from the RhoA family, which regulates the contractility of the cytoskeleton. Cells must have the ability to consume and redistribute chemical signals among their neighbors (gray arrows in Figure 10). These processes can be described by the following system of equations:

(9) $$J_{i_{j}}=\delta l_{ij}\left(C_{i}-C_{j}\right),\;\frac{dC_{i}}{dt}=\sum_{j=1}^{n_{0}}J_{i_{j}}-\beta C_{i}.$$

The left equation describes the intercellular diffusion of chemical signals between neighboring cells of the *i*th cell (see Figure 10). Here, *δ* is the diffusion coefficient, and **C** is the vector of concentrations of chemical signals involved in cell activity. The right equation describes the consumption and redistribution of these signals, where the flux is summed over the number of neighbors *n*_0_ of the *i*th cell, and *β* is the signal degradation coefficient.

In addition to the mentioned equations, we can take into account the chemo‐mechanical interaction of real tissues. This becomes possible due to the introduced system of Equation (9). By establishing a simple relationship of Equation (10) between mechanical parameters and chemical parameters, we have the opportunity to simulate the evolution of living tissues, which are essentially chemo‐elastomers.

(10) $$\begin{array}{l} \eta =\eta \left(C_{i}\right),\;\mu =\mu \left(C_{i}\right),\;\sigma =\sigma \left(C_{i}\right),\;\phi =\phi \left(C_{i}\right),\\ k=k\left(C_{i}\right),\;p_{0}=p_{0}\left(C_{i}\right),\;d_{\mathrm{int}}=d_{\mathrm{int}}\left(C_{i}\right) \end{array}.$$

The numerical solution of the system of equations ((1), (2), (3), (4), (5), (6), (7), (8), (9), (10)) was performed using the explicit Euler method, whose stability was ensured by a sufficiently small time step ∆*t* = 0.005. The simulation of the molecular field dynamics governed by Equation (9) was synchronized with the calculation of the mechanical movement (Equations 6 and 7) of epithelial tissue cells. The initial configuration in all cases consisted of a single hexagonal cell. Table 1 provides the fixed values of the control parameters, which were used in all numerical computations. It should be noted that all quantities are presented in arbitrary units. This means that we have the ability to relate each parameter of the system of equations ((1), (2), (3), (4), (5), (6), (7), (8), (9), (10)) to real values, depending on the specific problem being solved.

**TABLE 1:** The parameters of the mathematical model.

*μ*	*η*	*σ*	*ϕ*	*A*_0_	*l*_0_	*α*_0_	*k*	*F*_0_	*p*_0_	*q*	*d*_int_	*δ*	*β*
Changeable parameters				3√3/2	1.0	π/3	0.5	0.1	5 × 10^−5^	1.4	0.15	1.0	0.04

It should be noted that the proposed model allows tracking the complete history of all changes in each cell within the considered ensemble, and the total number of cells in the ensemble is only limited by the computational power of the computer. The developed discrete mathematical model, despite its simplicity, enables accurately describing the functional unit of the tissue—the cell, tracking its dynamics in space and faithfully reproducing cellular processes such as cell division, intercalation, and exchange of chemo‐mechanical signals. All of this, in turn, opens up the possibility of describing processes such as wound healing [7], tissue development in scaffold pores [28], phenotypic switching and cell migration [29], morphogenesis [11], and development of malignant formations [10, 29, 31, 32] with a certain level of accuracy.

## AUTHOR CONTRIBUTIONS

**Ivan Krasnyakov**: Conceptualization; data curation; formal analysis; funding acquisition; software; writing—original draft; writing—review and editing.

## CONFLICT OF INTEREST STATEMENT

The author Krasnyakov Ivan declares that there is no conflict of interest.

## ETHICS STATEMENT

This article does not contain any studies with human or animal subjects performed by the author.

## Supporting information

Figure S1

Figure S2

Figure S3

## Data Availability

The supplementary materials can be found online with this article.
