# Supplementary figures and images for: Mathematical modeling of evolution of cell networks in epithelial tissues
Source: Quant Biol. 2024 Jul 7;12(3):286–300. doi: 10.1002/qub2.62 (PMC12806389; doi:10.1002/qub2.62)

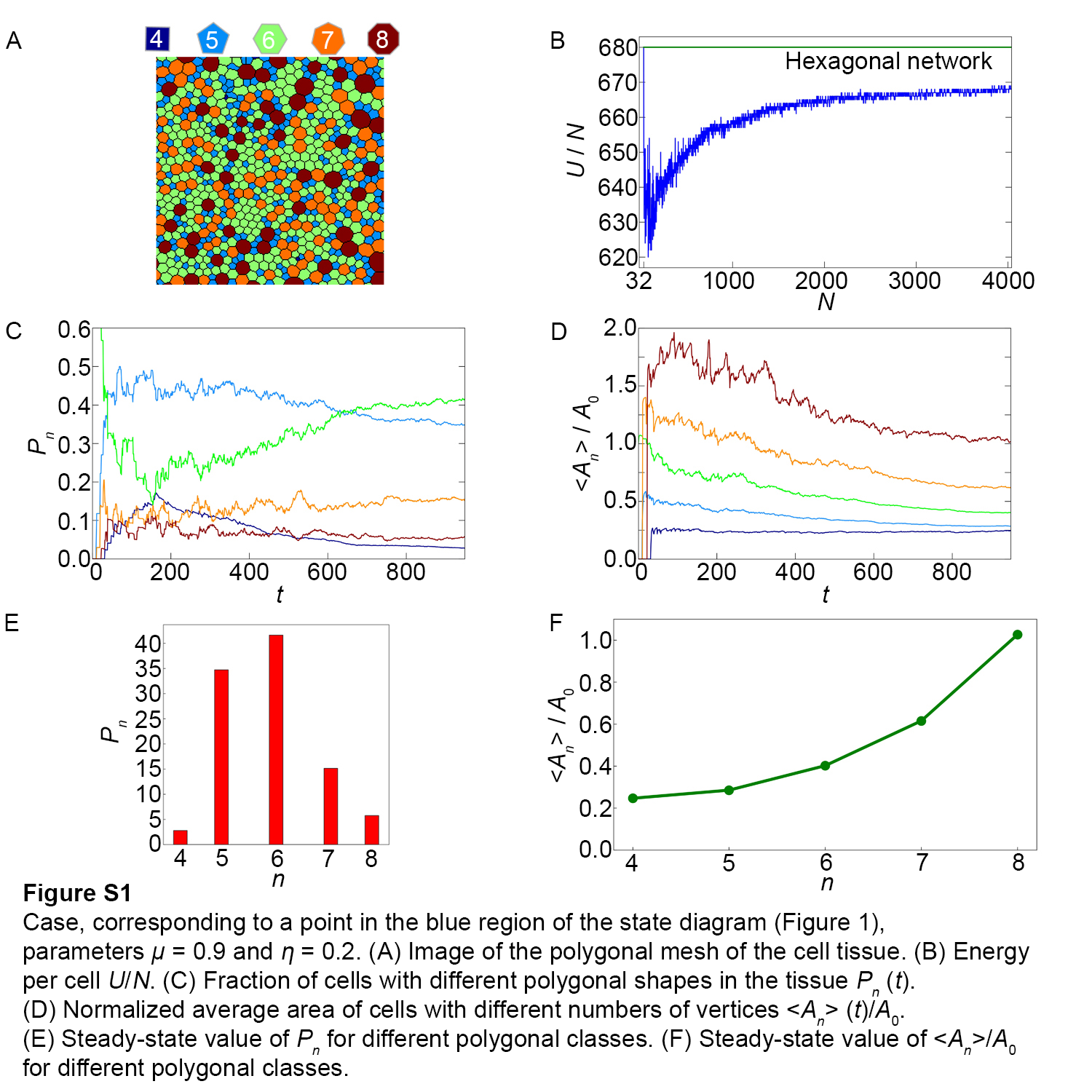

Supplement: Supplementary file 1 — Figure S1 [file QUB2-12-286-s002.tif]

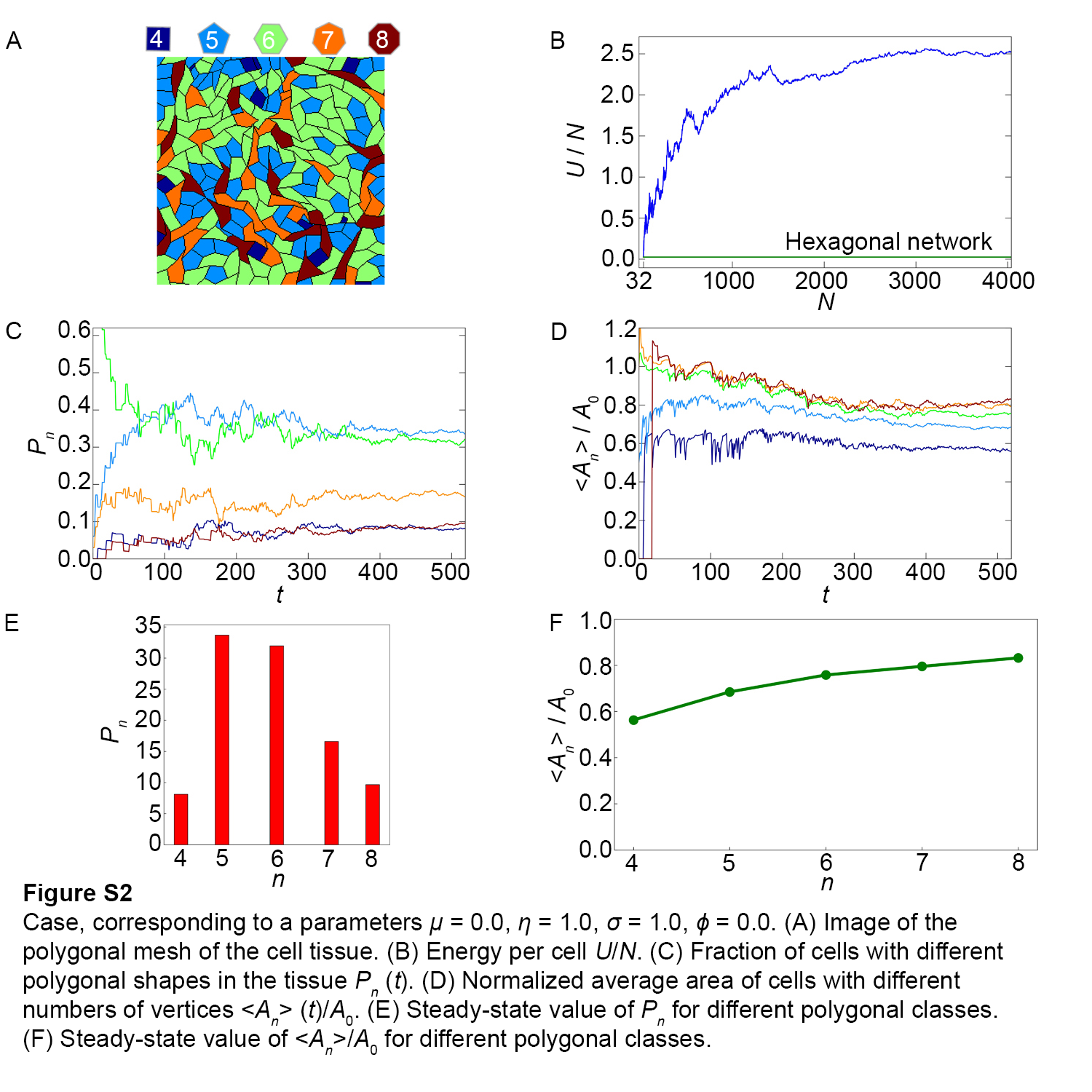

Supplement: Supplementary file 2 — Figure S2 [file QUB2-12-286-s003.tif]

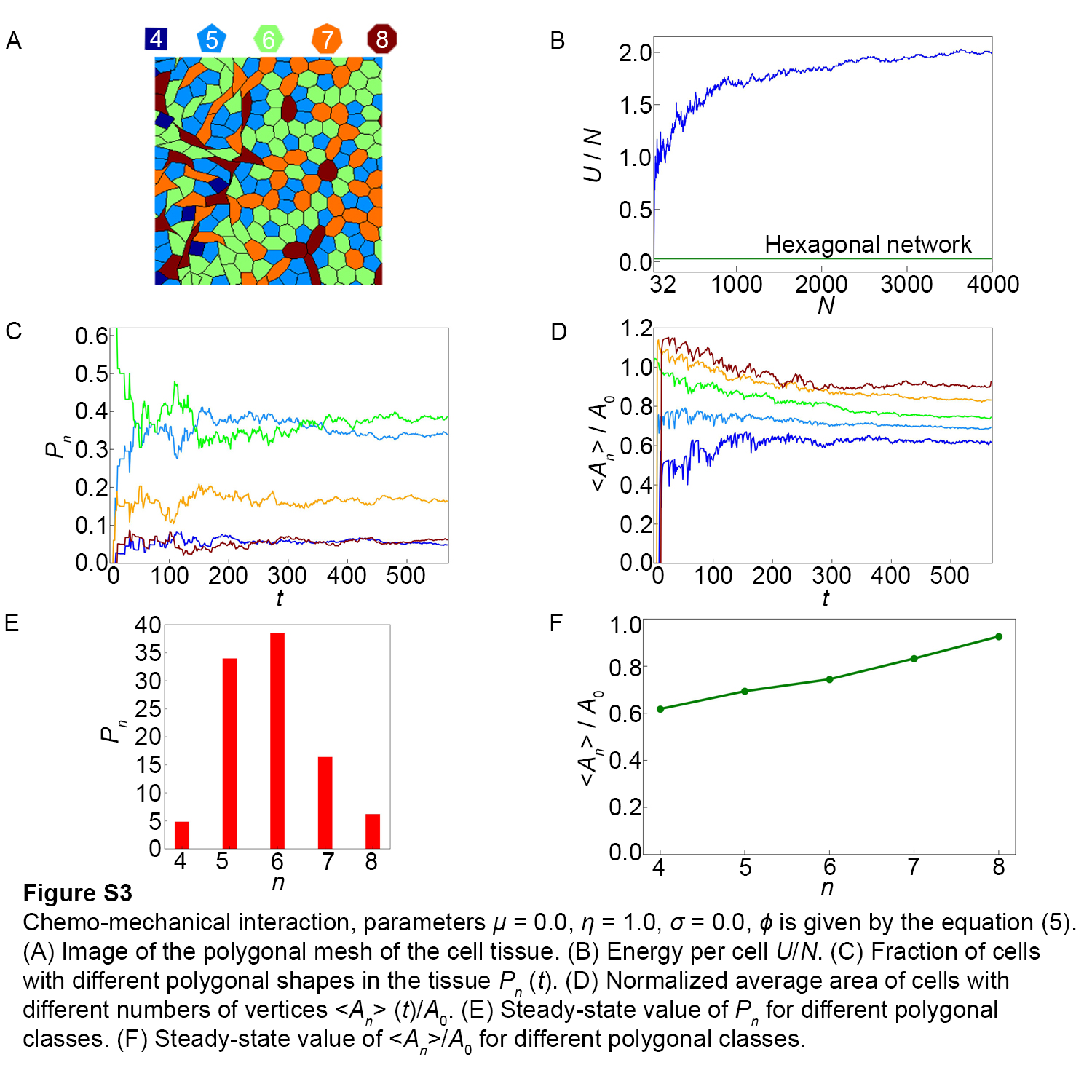

Supplement: Supplementary file 3 — Figure S3 [file QUB2-12-286-s001.tif]
